# Phase II Protocol on the Safety and Nutritional Status of Postoperative Enteral Nutrition Using HINEX® E-gel LC in Patients with Esophageal Cancer Undergoing Minimally Invasive Esophagectomy

**DOI:** 10.24546/0100495984

**Published:** 2025-05-30

**Authors:** TAKASHI KATO, HIRONOBU GOTO, MICHIKO TAKAHASHI, YASUFUMI KOTERAZAWA, RYUICHIRO SAWADA, HITOSHI HARADA, NAOKI URAKAWA, HIROSHI HASEGAWA, SHINGO KANAJI, KIMIHIRO YAMASHITA, TAKERU MATSUDA, TARO OSHIKIRI, YOSHIHIRO KAKEJI

**Affiliations:** 1 Division of Gastrointestinal Surgery, Department of Surgery, Kobe University Graduate School of Medicine, Kobe, Japan; 2 Department of Nutrition, Kobe University Graduate School of Medicine, Kobe, Japan; 3 Department of Gastrointestinal Surgery and Surgical Oncology, Graduate School of Medicine, Ehime University, Ehime, Japan

**Keywords:** Postoperative enteral nutrition, Minimally invasive esophagectomy, Oligomeric formula nutrient

## Abstract

**BACKGROUND:**

Enteral nutrition therapy in the early postoperative period is essential for patients with esophageal cancer following esophagectomy. HINEX® E-gel LC is an oligomeric formula with various nutrients, including dietary fiber pectin. It is expected to reduce symptoms, such as diarrhea, resulting in improved adherence and a decreased rate of weight loss. However, no reports have examined the adherence to or efficacy of nutritional support therapy using HINEX® E-gel LC after esophagectomy.

**METHODS:**

This is a single-institution, single-arm phase II trial. We plan to recruit 32 patients with esophageal cancer who have undergone minimally invasive esophagectomy (MIE) and place an enteral feeding tube. Enteral feeding is to be initiated on the second postoperative day, and the patient is to remain on enteral feeding at 300 kcal/day after discharge. The primary endpoint is adherence to enteral nutrition with HINEX® E-gel LC for up to 2 months postoperatively. The secondary endpoint is the association between the adherence to HINEX® E-gel LC and each nutritional parameter, such as hematological examination, body weight, and psoas muscle.

**DISCUSSION:**

Although enteral nutrition is usually initiated in the early postoperative period after esophagectomy, the use of fat-containing digestive agents in the early postoperative period is a novel approach. To the best of our knowledge, this study is the first to evaluate the safety and nutritional status of early postoperative enteral nutrition using fat-containing digestive agents after MIE. Efficient nutritional support therapy using fat-containing digestive agents is expected to be especially useful after esophagectomy when oral intake is significantly decreased.

## INTRODUCTION

Malnutrition due to decreased nutritional intake is one of the most common postoperative disorders following esophagectomy for esophageal cancer; it is a prognostic factor for esophageal cancer [1–3]. Sarcopenia, a condition in which skeletal muscle mass decreases with age, is associated with poor postoperative outcomes [4, 5].

Enteral nutritional therapy in the early postoperative period is widely used to prevent malnutrition after esophagectomy. However, adherence to postoperative nutritional supplementation remains an issue. Nutritional support therapy with Lacol® NF, a polymeric formula, has been reported to maintain adherence to dosing, which contributed to lower weight loss rates for patients with gastric cancer [6]. In contrast, abdominal discomfort and diarrhea caused by oral nutritional supplementation affect adherence in patients who undergo gastrectomy [7, 8].

HINEX® E-gel LC is an oligomeric formula with various nutrients adjusted with reference to the “Dietary Reference Intakes for Japanese, 2020” [9]. Compared with conventional nutritional supplements, the inclusion of dietary pectin is expected to reduce symptoms such as diarrhea, resulting in improved adherence and a lower rate of weight loss. In addition, compared to the component nutrients, dissolving the drug is not required, and it is easy to use. However, no reports have examined the adherence to or efficacy of nutritional support therapy with this formulation after esophagectomy.

This study aimed to evaluate the adherence to HINEX® E-gel LC for patients who underwent minimally invasive esophagectomy (MIE) for esophageal cancer.

## METHODS AND DESIGN

### Hypothesis

This study hypothesizes that HINEX® E-gel LC in an oligomeric formulation can be safely administered as enteral nutrition after MIE.

### Study design, endpoints, and period

This is a single-center, single-arm, phase II trial to evaluate the safety and nutritional status of postoperative enteral nutrition using an oligomeric formula nutrient (HINEX® E-gel LC) in patients with esophageal cancer undergoing MIE. The primary endpoint is adherence to enteral nutrition with HINEX® E-gel LC for up to 2 months postoperatively. The secondary endpoint is the association between the adherence to HINEX® E-gel LC and each nutritional parameter, such as hematological examination, body weight, and psoas muscle. The expected total duration of patient enrollment is 2 years, and the follow-up period is 6 months.

### Patient selection and enrollment criteria

#### Inclusion criteria

The inclusion criteria will be as follows: (i) age is over 18 years old at the registration date; (ii) the clinical stage was I, II, or III according to the Japanese Classification of Esophageal Cancer, 12th edition (based on esophagogastroduodenoscopy, computed tomography, and positron emission tomography); (iii) an Eastern Cooperative Oncology Group performance status of 0, 1, or 2; (iv) oral intake is feasible preoperatively; (v) esophagectomy is expected within 60 days from the date of registration; (vi) oral intake is expected for 3 months after esophagectomy; and (vii) written informed consent obtained.

#### Exclusion criteria

The exclusion criteria will be as follows: (i) history of hypersensitivity to any element of this nutritional supplement; (ii) allergies to any of the material (especially soy and gelatin) in the test nutritional product, HINEX® E-Gel LC; (iii) difficulty in obtaining consent because of dementia or psychiatric diseases that require treatment; (iv) use of steroids or immunosuppressive drugs; (v) infectious disease requiring systemic treatment; and (vi) any other information deemed inappropriate by the investigator.

#### Discontinuing criteria

Protocol treatment will be discontinued if the following criteria are met: (i) requesting treatment discontinuation because of illness or other reasons; (ii) fasting >30 days after surgery; (iii) undergoing non-curative resection; (iv) recurrence of the primary disease during the study period; (v) death during protocol treatment; and (vi) withdrawal of patient consent.

Even after discontinuation of protocol treatment, unless participation in the study is discontinued, investigation and observation as provided for in the research will be conducted until the end of the follow-up period.

### Sample size

In this trial, the sample size for analysis was 32 patients. In a phase III clinical trial of Twinline® [10], a digestive-form nutritional supplement, 167 patients with gastrointestinal diseases requiring enteral nutrition were participated. Of the 167 patients, 8 had dose reductions due to adverse effects and 13 discontinued due to adverse effects under the condition that the dosing period was 5 days or longer. The main adverse events that occurred in this study were diarrhea, abdominal pain, and abdominal distention. Of the 167 patients, 146 were able to complete the 5-day dosing, or an estimated 87.4%. In this trial we have planned, we expect the completion rate to be at 2 months, which is the typical duration of feeding tube use in clinical practice. If 23 of the 25 cases in the analysis were completed, the completion rate is estimated to be 92.0%, with 95% confidence intervals of 74% and 99%. This setting was made based on the assumption that the use of HINEX® E-gel LC would have an additional effect on the conventional clinical trial of Twinline®. After examining 25 patients and estimating the exclusion of approximately five patients because of complications such as anastomotic leakage and the discontinuation of approximately two patients due to cancer recurrence, the sample size for this trial was calculated to be 32 patients. The above settings were also estimated based on the number of operations per year at our hospital, as we estimated the duration of the trial to be approximately 1 year. The sample size was also calculated using JMP® version 14.2 (SAS Institute Inc., Cary, NC, USA).

### Treatment

**Figure 1:**
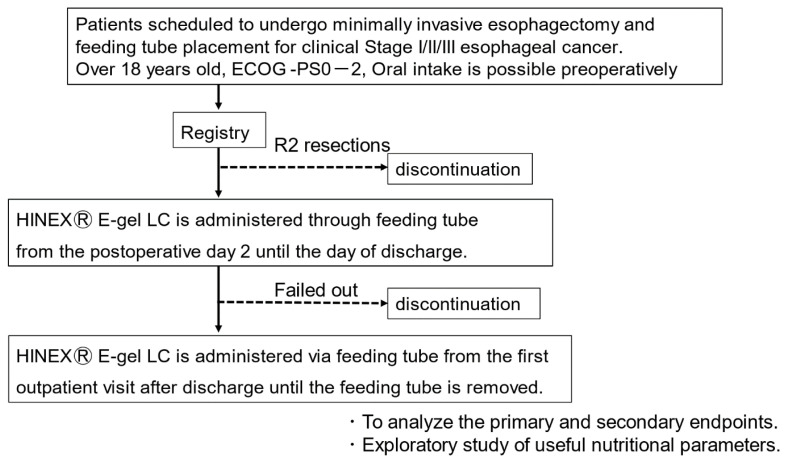
Trial design

The eligibility assessment for this study is shown in Figure 1. All patients underwent MIE, including robotic surgery, in the prone position. After the thoracic procedure, gastric conduit reconstruction via the retrosternal route and feeding tube placement is performed, and a feeding tube is placed. Enteral feeding is initiated on the second postoperative day, and the amount of enteral feeding is gradually increased while observing the abdominal symptoms. Oral intake begins 1 week after esophagectomy, and the amount of enteral feeding is tapered off. After discharge from the hospital, the patient will continue to receive enteral feeding of 300 kcal/day at home, and the tube will be removed at an outpatient hospital when oral intake is considered sufficient. For enteral nutrition support at home, we will ask the patients to write down what they have eaten and weight changes in order to collect accurate information, which we will use to provide nutritional support. Since enteral nutrition for approximately 2 months after esophagectomy is common in previous studies, we will observe the completion rate at 2 months in this study as well. The purpose of this study is to investigate patient compliance with administration of the nutritional products; therefore, a self-interruption of more than 2 weeks is not considered a protocol deviation, but it will be recorded as a failure to the study.

### Participant timeline

The schedule for conducting the observations, examinations, and evaluations of the participants is presented in Table I. The investigator will conduct observations and examinations according to the schedule.

### Follow-up and assessment

The percentage of patients who can continue taking HINEX® E-gel LC daily for up to 2 months after surgery will be evaluated. The occurrence of illness and other symptoms will be evaluated as part of the safety assessment (gastrointestinal symptoms related to protocol treatment: diarrhea, constipation, vomiting, and others) and the duration of use of HINEX® E-Gel will be evaluated. The Common Terminology Criteria for Adverse Events (CTCAE) ver. 5.0 will be used to evaluate illness.

The following secondary endpoints will be evaluated as nutritional indicators until 6 months postoperatively: (i) Eastern Cooperative Oncology Group (ECOG)-Performance Status (PS) (2, 3, and 6 months postoperatively); (ii) nutritional index parameters based on blood samples (preoperatively, 2, 3, and 6 months postoperatively; total protein [TP], serum albumin [ALB], total lymphocyte count, total cholesterol, transthyretin, hemoglobin [Hb], C-reactive protein [CRP]); (iii) body weight loss rate (BWL) at 2, 3, and 6 months postoperatively (%BWL = Preoperative weight | Postoperative weight/Preoperative weight × 100 [%]); (iv) skeletal muscle mass using computed tomography (CT) images (preoperatively and 6 months postoperatively); and (v) measurement of body composition and muscle mass using bioelectrical impedance analysis (BIA) (preoperatively, 2, 3, and 6 months postoperatively).

Additionally, patient background (sex, age, height, weight, medical history, family history, drinking history, and smoking history) and surgical information (operative procedure, operative time, blood loss, and postoperative complications) were evaluated.

### Statistical methods

The primary analysis will be to calculate the completion rate of enteral nutrition at 2 months postoperatively, which is the primary endpoint of this clinical study, and to estimate the 95% confidence interval. Efficacy will be analyzed as a secondary endpoint.

### Evaluation and reporting of adverse events

The physician will document all diseases that occur from the time the trial product is used to the date of completion of its use in a case report form and submit it to the research office. The investigator will observe the patients until the disease is resolved or until two weeks after the end of their participation in the clinical research.

### Ethics approval and consent to participate

This clinical research will be conducted in compliance with the Declaration of Helsinki, the Clinical Research Act, the Conflict of Interest Management Plan for this clinical research, and other relevant laws and regulations. This clinical study has been registered on jRCT, a public clinical trials registry site and approved by the Ethics Committee of Kobe University (No. CRB5180009).

## DISCUSSION

Surgical resection is a standard treatment for patients with esophageal cancer. However, esophagectomy is associated with higher morbidity and mortality rates than other gastrointestinal surgeries. Recently, MIE has become the standard surgical procedure for esophageal cancer worldwide and has been reported to reduce postoperative respiratory complications, shorten hospital stays, and improve quality of life compared to open esophagectomy [11]. Therefore, overcoming postoperative malnutrition caused by decreased nutritional intake after MIE is important.

Sarcopenia is associated with poor postoperative outcomes in various cancer types [4]. Therefore, the completion rate of enteral nutrition after surgery may be related to improved long-term postoperative outcomes. Hinex® E-Gel LC used in this study is a digestive-form liquid food containing dietary fiber pectin. This food was designed considering hydration, and the amount of water that could be consumed per 100 kcal was adjusted to 110 mL. Although enteral nutrition is usually initiated in the early postoperative period after esophagectomy, the use of fat-containing digestive agents in the early postoperative period is a novel approach. The novelty of this study is the use of fat-containing digestive-form in the early postoperative period. To the best of our knowledge, this study is the first to evaluate the safety and nutritional status of early postoperative enteral nutrition using fat-containing digestive agents after MIE. Efficient nutritional support therapy using fat-containing digestive agents is expected to be especially useful after esophagectomy when oral intake is significantly decreased.

Long-term enteral nutrition after MIE reduces skeletal muscle loss [12], while placement of an enterostomy in the jejunum increases the risk of bowel obstruction [13]. In this trial, we planned gastric conduit reconstruction through the retrosternal route and feeding tube placement from the antrum of the gastric tube. In addition, a round ligament of the liver is interposition, and a tube is fixed to the abdominal wall to prevent peritonitis after removing the feeding tube [14, 15].

Through this phase II trial, we aim to determine the safety and nutritional status of postoperative enteral nutrition using the oligomeric formula nutrient HINEX® E-gel LC in patients with esophageal cancer undergoing MIE.

## Figures and Tables

**Table I:** Schedule for observations, examinations, and evaluations of participants

Trial periods	Pre-operative	Perioperative/Postoperative				Follow-up			At the time of discontinuation of the trial
		Time of admission	POD 0	POD 2	POD 28	2 months	3 months	6 months	
The time window for a visit (days)	−60	−7	0	+7	±21	±21	±30	±30	
Informed consent	●								
Registry	●								
Feeding from HINEX® E-gel LC				●		●			
Confirmation of feeding (Primary endpoint)				●		●	●	●	
ECOG-PS (Secondary endpoint)	●					●	●	●	
Measuring body weight (Secondary endpoint)	●					●	●	●	
Laboratory sampling (Secondary endpoint)	●	●				●	●	●	
Computed tomography (Secondary endpoint)	●							●	
Bioelectrical impedance analysis (Secondary endpoint)	●					●	●	●	
Observation of diseases			●	●					●
Outpatient nutritional guidance	●				●		●	●	
Check the nutrition handbook						●	●	●	●

ECOG, Eastern Cooperative Oncology Group; PS, Performance status; POD, Postoperative days.
